# Fructose Consumption Affects Placental Production of H_2_S: Impact on Preeclampsia-Related Parameters

**DOI:** 10.3390/nu16020309

**Published:** 2024-01-20

**Authors:** Madelín Pérez-Armas, Elena Fauste, Cristina Donis, Silvia Rodrigo, Lourdes Rodríguez, Juan J. Álvarez-Millán, María I. Panadero, Paola Otero, Carlos Bocos

**Affiliations:** 1 Facultad de Farmacia, Universidad San Pablo-CEU, CEU Universities, Montepríncipe, 28668 Boadilla del Monte, Madrid, Spain; m.perez170@usp.ceu.es (M.P.-A.); elena.faustealonso@ceu.es (E.F.); c.donis@usp.ceu.es (C.D.); s.rodrigoduran@gmail.com (S.R.); lourdesrbilbao@gmail.com (L.R.); ipanade@ceu.es (M.I.P.); paotero@ceu.es (P.O.); 2 CQS Lab, 28521 Rivas Vaciamadrid, Madrid, Spain; jamillan@cqslab.com

**Keywords:** fructose, pregnancy, fetal programming, H_2_S, placenta, preeclampsia

## Abstract

H_2_S, a gasotransmitter that can be produced both via the transsulfuration pathway and non-enzymatically, plays a key role in vasodilation and angiogenesis during pregnancy. In fact, the involvement of H_2_S production on plasma levels of sFLT1, PGF, and other molecules related to preeclampsia has been demonstrated. Interestingly, we have found that maternal fructose intake (a common component of the Western diet) affects tissular H_2_S production. However, its consumption is allowed during pregnancy. Thus, (1) to study whether maternal fructose intake affects placental production of H_2_S in the offspring, when pregnant; and (2) to study if fructose consumption during pregnancy can increase the risk of preeclampsia, pregnant rats from fructose-fed mothers (10% *w*/*v*) subjected (FF) or not (FC) to a fructose supplementation were studied and compared to pregnant control rats (CC). Placental gene expression, H_2_S production, plasma sFLT1, and PGF were determined. Descendants of fructose-fed mothers (FC) presented an increase in H_2_S production. However, if they consumed fructose during their own gestation (FF), this effect was reversed so that the increase disappeared. Curiously, placental synthesis of H_2_S was mainly non-enzymatic. Related to this, placental expression of Cys dioxygenase, an enzyme involved in Cys catabolism (a molecule required for non-enzymatic H_2_S synthesis), was significantly decreased in FC rats. Related to preeclampsia, gene expression of sFLT1 (a molecule with antiangiogenic properties) was augmented in both FF and FC dams, although these differences were not reflected in their plasma levels. Furthermore, placental expression of PGF (a molecule with angiogenic properties) was decreased in both FC and FF dams, becoming significantly diminished in plasma of FC versus control dams. Both fructose consumption and maternal fructose intake induce changes in molecules that contribute to increasing the risk of preeclampsia, and these effects are not always mediated by changes in H_2_S production.

## 1. Introduction

Fructose consumption, a sweetener used in the food industry, either in the form of sucrose or high-fructose corn syrup (HFCS), has experienced a rapid increase for several reasons in the last decades. Fructose has a higher sweetening power and a lower glycemic index when compared to glucose [1,2]. Consequently, fructose has been proposed as an alternative to glucose for diabetic patients. However, several studies, including ours, have confirmed that consumption of this sugar is associated with a greater risk of obesity, cardiovascular diseases, metabolic syndrome, decreased insulin sensitivity, hypertriglyceridemia, and an increase in the production of reactive oxygen species [3,4,5,6]. 

During pregnancy, the fetal environment to which the fetus is exposed significantly influences the risk of certain diseases in adult life, and it is widely recognized that the mother’s health is a key factor influencing the correct development of the offspring. Fetal programming is an adaptative process that occurs during gestation [7,8], through which complications such as gestational diabetes, maternal stress, or poor nutrition can lead to phenotypic modifications in the fetus, resulting in changes in metabolism and an increased susceptibility to chronic diseases in adulthood. The placenta plays a crucial role in this process [9] since it is responsible for nutrients exchange between the mother and the fetus, leading to promoting a fetal plasticity that allows the fetus to adapt to the prenatal environment. This fetal adaptation produces epigenetic changes that persist after birth, and even remain in adulthood. 

**Figure 1:**
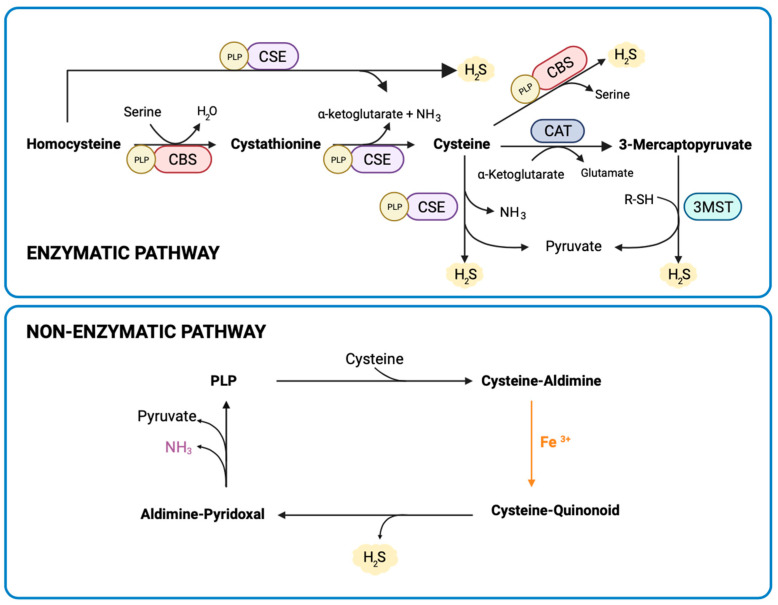
Enzymatic (transsulfuration pathway) and non-enzymatic routes to generate H_2_S in placenta. Cystathionine beta-synthase (CBS); cystathionine gamma-lyase (CSE); 3-mercaptopyruvate sulfotransferase (3MST); PLP: Pyridoxal 5′-phosphate. Created with BioRender.com.

Hydrogen sulfide (H_2_S) is an endogenous gasotransmitter involved in many physiological and pathological processes, particularly in the inflammatory response, where it exerts an anti-inflammatory effect in a large number of tissues [10]. Homocysteine is a sulfur-containing amino acid generated from methionine metabolism. Homocysteine accumulation has been related to cardiovascular diseases. Its elimination occurs mainly through two metabolic pathways, transsulfuration, and remethylation [11]. The transsulfuration pathway plays a fundamental role in sulfur metabolism and redox regulation of cells. Through this metabolic route, homocysteine is transformed into cysteine, and H_2_S is generated as a byproduct. Three enzymes are involved in this pathway: cystathionine-β-synthase (CBS), cystathionine-γ-lyase (CSE), and 3-mercapto pyruvate sulfotransferase (3MST) (Figure 1).

Another way to generate H_2_S is through a non-enzymatic reaction process described by Yang et al. [12]. This reaction path occurs by using cysteine as a substrate and it also requires vitamin B6, as well as pyridoxal phosphate (PLP) and iron (Figure 1). It is worth noting that the production of H_2_S through the non-enzymatic pathway is not as strictly regulated as the enzymatic pathway, and its regulation is still unknown. 

Based on its vasodilatory role, several studies have linked H_2_S to key processes in pregnancy [13,14] such as labor, or pregnancy complications like preeclampsia [15,16]. Preeclampsia is a condition characterized by increased blood pressure and signs of damage to other organs, most commonly liver and kidneys, that causes the death of over 50,000 pregnant women worldwide every year [17]. Moreover, it is a major cause of fetal morbidity and mortality. Unfortunately, delivery of the fetus is the only effective treatment available [18], which raises the importance of a better understanding of the pathogenesis, including the risk factors that can promote the development of this disease. 

Furthermore, some studies have shown that circulating levels of soluble fms-like tyrosine kinase-1 (sFLT1), a soluble variant of the vascular endothelial growth factor receptor (VEGFR1), are increased in patients with preeclampsia [19]. sFLT1 is able to bind and block the vascular endothelial growth factor (VEGF) and the placental growth factor (PGF), two proangiogenic proteins that are found in lower levels in this disease. The cause of this diminution is the increased presence of sFLT1, since it acts as an antagonist, preventing the proangiogenic signaling induced by these two molecules [20]. 

In addition, women diagnosed with preeclampsia present a decrease in plasma H_2_S levels, linking this decrease to a diminution in CBS and CSE enzymatic activities [21]. Moreover, Wang et al. have shown that an augmented CSE-mediated H_2_S production inhibits the release of antiangiogenic factors (sFLT1 and soluble endoglin (sEng)) [22]. However, there is still limited knowledge on how H_2_S influences sFLT1 and sEng production [23].

Interestingly, in previous studies, we demonstrated that fructose intake produced a diminution in the CSE liver expression and, consequently, in the production of H_2_S [24,25]. Therefore, maternal fructose intake could reduce placental H_2_S production affecting, thus, antiangiogenic proteins release and, accordingly, promoting preeclampsia or other risk situations for pregnancy. Given fructose consumption during pregnancy is allowed, the aims of the present study were: (1) to study whether fructose consumption affects placental H_2_S production; (2) to study whether maternal fructose intake affects H_2_S synthesis in the offspring, when pregnant; and (3) to determine whether fructose consumption during pregnancy can increase the risk of preeclampsia.

## 2. Materials and Methods

### 2.1. Animals and Experimental Design

A model of maternal liquid fructose consumption was established in rats following previously outlined procedures [26,27]. Female Sprague Dawley rats weighing between 200–240 g were fed a standard rat chow diet ad libitum (B&K Universal, Barcelona, Spain), and housed in conditions with controlled light and temperature (12 h light–dark cycle; 22 ± 1 °C). The Ethical Committee for Animal Experimentation of the University San Pablo-CEU and the Autonomous Government of Madrid approved the experimental protocol (ref. number 10/206458.9/13). Pregnant animals (F0 generation) were randomly assigned to a control group, a fructose-fed group (fructose), and a glucose-fed group (glucose) (6–7 rats per group). Throughout gestation, fructose and glucose were administered as a 10% (wt/vol) solution in drinking water. Control animals did not receive any additional sugar. On day 21 of gestation, food was withdrawn at 8 a.m., and two hours later, pregnant rats were euthanized. Plasma was obtained, aliquoted, and stored at −80 °C. Placentas were placed in liquid nitrogen and preserved at −80 °C for subsequent analysis.

A different group of pregnant rats was provided unrestricted access to a standard rat chow diet (Teklad Global 14% Protein Rodent Maintenance Diet, Envigo, Madison, WI USA), and were housed in conditions with controlled light and temperature (12 h light–dark cycle; 22 ± 1 °C). The experimental protocol was approved by the Ethical Committee for Animal Experimentation of the University San Pablo-CEU and the Autonomous Government of Madrid (ref. numbers 10/206458.9/13 and 10/042445.9/19). The experimental procedures applied to the pregnant rats (F0 generation) were consistent with those previously detailed [26]. Briefly, pregnant rats were randomly assigned to either a control group (receiving no supplementary sugar in drinking water) or a fructose-supplemented group (fructose 10% wt/vol in drinking water) throughout gestation (5 rats per group). After giving birth, each suckling litter was standardized to nine pups. Following delivery, both mothers and their pups were provided with water (with no supplementation) and food ad libitum.

At 21 days of age, female offspring (F1 generation) were separated from their mothers and maintained on a standard rat chow diet and water without additives [27]. Upon reaching 8 weeks of age, they were mated. Subsequently, pregnant rats from control mothers were sustained on solid pellets and provided with tap water devoid of additional sugar during gestation, constituting the CC group. Conversely, pregnant rats from mothers exposed to fructose during pregnancy were randomly divided into two groups. Animals within each experimental group were born to different dams to minimize the ‘‘litter effect’’. Thus, one set of pregnant rats was fed a standard rat chow diet and had access to water without any additives (FC group), while the other half of pregnant rats consumed fructose at a concentration of 10% wt/vol in drinking water (FF group) throughout gestation (5 rats per group). Thus, three distinct experimental groups were established: the first letter denoting whether the mothers (F0 generation) had been provided with tap water during pregnancy (C, control) or water containing fructose (F, fructose); and the second letter indicating whether the offspring (F1 generation) received fructose (F) or not (C) during their own pregnancy.

On the 21st day of gestation, pregnant rats were euthanized at 10:00. Two hours prior to the sacrifice, food was withheld. Plasma was obtained, divided into aliquots, and preserved at −80 °C. Placentas were placed in liquid nitrogen and preserved at −80 °C for subsequent analysis.

### 2.2. Plasma Determinations

Specific ELISA kits were used to measure sFLT-1 (R&D Systems, Biotechne, Minneapolis, MN, USA) and PGF (Cusabio Biotech Co., Ltd., Wuhan, China) in plasma samples following the manufacturer instructions.

### 2.3. Measurement of Placental H_2_S Synthesis

Total production of H_2_S in the tissue was determined using the lead sulfide method as previously described with modifications [28]. Briefly, tissue homogenates were prepared in phosphate-buffered saline (PBS) and homogenized using a TissueLyser^®^ (Qiagen, Germantown, MD, USA). Protein content in the supernatant was measured using the Pierce^®^ BCA Protein Assay kit (ThermoFisher Scientific, Waltham, MA, USA).

Once the protein content in the samples was determined, an amount of sample corresponding to 300 μg of protein was incubated at 37 °C for 3 h in the presence of 40 mM L-Cys (Sigma-Aldrich, St. Louis, MO, USA) and 2 mM pyridoxal 5′-phosphate (PLP, Sigma-Aldrich, MO, USA) on 96-well plates covered with a lead acetate (II) membrane. To prepare these membranes, Whatman n◦ 2 paper was soaked into 20 mM lead acetate (Sigma-Aldrich, MO, USA) and vacuum dried. A calibration curve from 0 to 1000 nM of NaHS (Fluorochem, Glossop, UK) was performed for each membrane and treated under the same conditions as samples. When dots of lead sulfide precipitated were detected were densitometered using a BioRad Densitometer G-800 (BioRad, Hercules, CA, USA).

To determine the non-enzymatic production of H_2_S, the protocol described above was modified and two different procedures were carried out [12]: (1) a pretreatment of the samples with proteinase K, and (2) D-cysteine was used as substrate. In the first case, PBS homogenates of placenta were treated with 100 μg/mL of proteinase K (Sigma, USA) for 1 h at 37 °C. The second modification involved using 40 mM D-cysteine (Fluorochem, UK) instead of L-cysteine. Unlike the L-form, D-Cys is not used by the transsulfuration pathway enzymes.

Finally, to measure the production of H_2_S exclusively via the enzymatic pathway, homogenates in PBS were treated with 150 mM EDTA (Sigma, USA). EDTA is a metal ion chelator, and iron ions are necessary for the non-enzymatic synthesis of H_2_S. In this case, the reaction needed to be incubated for 24 h to observe the appearance of lead sulfide precipitates.

### 2.4. RNA Extraction and Gene Expression Determination by qPCR

Total RNA was isolated from placenta using Ribopure (Invitrogen, ThermoFisher Scientific, Waltham, MA, USA). RNA was subjected to DNase I treatment using Turbo DNA-free (Invitrogen, ThermoFisher Scientific, Waltham, MA, USA), and RNA integrity was confirmed by agarose gel electrophoresis. Afterwards, cDNA was synthesized by oligo(dT)-primed reverse transcription with Superscript II (Invitrogen, ThermoFisher Scientific, Waltham, MA, USA). qPCRs were performed using a CXF96^®^ Touch (Bio-Rad, Hercules, CA, USA). The reaction solution was carried out in a volume of 20 μL, containing 10 pmol of both forward and reverse primers, 10× SYBR Premix Ex Taq (Takara Bio Inc., Kusatsu City, Japan), and the appropriate nanograms of the cDNA stock. Rps29 was used as a reference gene for qPCR. The primer sequences were designed using Primer Blast (NCBI, Bethesda, MD, USA). Samples were analyzed in duplicate on each assay. qPCR efficiency and linearity were assessed by optimization of the standard curves for each target. The transcription was quantified with CFX Maestro 2.0 software (Bio-Rad, Hercules, CA, USA) using the efficiency correction method [29].

### 2.5. Statistical Analysis

Results were expressed as means ± S.E. Treatment effects were analyzed by one-way analysis of variance (ANOVA). When treatment effects were significantly different (*p* < 0.05), means were tested by Tukey’s multiple range test using SPSS (version 27) program. If the variance was not homogeneous, a post hoc Tamhane test was performed. Significant differences between the experimental groups are indicated by using different letters.

## 3. Results

### 3.1. Placental H_2_S Is Mainly Non-Enzymatically Produced

**Figure 2:**
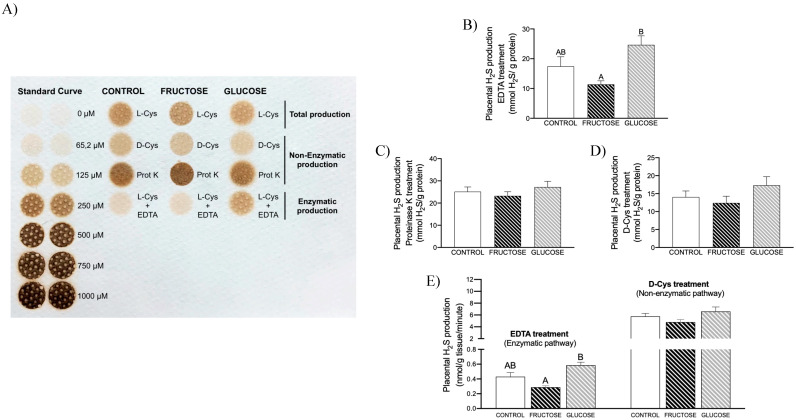
Carbohydrate (fructose or glucose) consumption during pregnancy affects H_2_S production in placenta. (**A**) Membrane showing lead acetate precipitates formed in the assay: Total H_2_S generation in the presence of L-Cys (after 3 h of incubation); enzymatic production of H_2_S after EDTA treatment (and 24 h of incubation); and non-enzymatic synthesis of H_2_S after proteinase K treatment or in the presence of D-Cys (after 3 h of incubation). Darker precipitates indicate higher H_2_S production in the tissue. Enzymatic (**B**) and non-enzymatic (proteinase K, (**C**)) or (D-Cys, (**D**)) placental H_2_S levels of control (empty bar), fructose-fed (grey bar), or glucose-fed (black bar) pregnant rats. (**E**) To compare the different H_2_S production rates between enzymatic and non-enzymatic pathways in placenta, different scales were used for the y axis before and after the break. Data are expressed as means ± S.E., *n* = 7 (control); 7 (fructose); and 6 (glucose) rats. Net H_2_S produced was expressed as nanomoles per gram of protein; H_2_S production rates were expressed as nanomoles per minute per gram of tissue. Values not sharing a common letter are significantly different (*p* < 0.05).

In previous studies, we have demonstrated that fructose intake reduces both liver expression of CSE and H_2_S production [24,25]. Placental H_2_S production from L-Cys also showed a trend to decrease in fructose-fed dams (70.78 ± 8.23, 65.72 ± 6.21, 72.32 ± 8.82 mmol/g protein for control, fructose-, and glucose-fed dams, respectively), without reaching statistical significance, in comparison to control rats. Curiously, however, CBS gene expression presented an increase in the placentas of fructose-fed pregnant rats, and CSE and 3MST gene expression did not show significant changes between the three experimental groups (Table S1). To help us to explain these discrepancies, and since another way to generate H_2_S exists [12] through a non-enzymatic process, we decided to study both the enzymatic and non-enzymatic pathways. Thus, as shown in Figure 2A, clear differences were observed in the intensities of lead acetate precipitates formed in the membrane when comparing total H_2_S generation in the presence of L-Cys, enzymatic production of H_2_S, and non-enzymatic synthesis of H_2_S. Moreover, to correctly evaluate these differences, we need to consider that different times of incubation were used in each case: 3 h for non-enzymatic and total production assays, in comparison to the 24 h required for the enzymatic test. Furthermore, clearly marked differences were observed between the three experimental groups. Thus, whereas enzymatic synthesis of H_2_S presented a decrease in placenta of fructose-fed pregnant rats that was significant when compared to glucose-fed mothers (Figure 2B, *p* = 0.007), non-enzymatic H_2_S production using both proteinase K and D-Cys (Figure 2C,D, respectively) did not show significant changes between the three experimental groups of pregnant rats. Nevertheless, the most remarkable finding observed here was the huge difference when comparing placental H_2_S production rates (which consider H_2_S generation as a function of incubation times), reaching values ten times higher in the non-enzymatic process production. (Figure 2E). Therefore, surprisingly, placental H_2_S production turned out to be mainly non-enzymatic.

### 3.2. Maternal Intake of Fructose Increases H_2_S Production in Placenta of Descendants

In a previous study, we demonstrate that maternal fructose intake produces changes in female descendants that alter their own pregnancy, leading to deleterious effects in their fetuses [27]. Since we found that fructose intake during pregnancy can directly affect the enzymatic production of H_2_S in placenta (Figure 2B,E), we were interested to know if maternal fructose intake can also induce epigenetic changes that would affect placental production of H_2_S in the offspring through a fetal programming mechanism. To achieve this goal, pregnant rats from fructose-fed dams subjected (FF) or not (FC) to a fructose intake were studied and compared to pregnant control rats (CC). 

**Figure 3:**
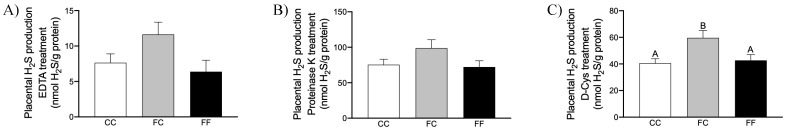
Maternal fructose intake affects placental H_2_S production of their descendants. Enzymatic (**A**) and non-enzymatic (proteinase K, (**B**)) or (D-Cys, (**C**)) placental H_2_S levels of 21-day-pregnant rats from control mothers (CC, empty bar), or pregnant rats from fructose-fed mothers subjected (FF, black bar) or not (FC, grey bar) to fructose intake throughout pregnancy. Data are expressed as means ± S.E., n = 5 (CC); 4 (FC); and 5 (FF) rats. Values not sharing a common letter are significantly different (*p* < 0.05).

Curiously, as shown in Figure 3, descendants of fructose-fed mothers that consumed water without additives (FC) presented an increase in both enzymatic (Figure 3A) and, mainly, non-enzymatic production of H_2_S (Figure 3B,C), an effect that was not observed in the progeny from fructose-fed mothers that consumed fructose during their gestation (FF). In fact, the increase in non-enzymatic H_2_S production from D-Cys found in FC dams was significantly higher versus CC and FF (*p* = 0.029 and *p* = 0.05, respectively). Interestingly, whereas maternal fructose consumption induces placental H_2_S synthesis in the offspring, fructose intake seems to produce the directly opposite effect: FF vs. FC (Figure 3C) and fructose-fed versus glucose-fed dams (Figure 2B). Therefore, fructose consumption leads to a diminution in placental H_2_S production.

Given that gene expression of enzymes involved in the transsulfuration pathway did not show significant changes between the three experimental groups (Table S2), and since we have demonstrated above that the enzymatic pathway is only a minor mechanism for H_2_S generation in the placenta, we focused on elucidating the mechanisms responsible for the changes observed in the non-enzymatic pathway of H_2_S production. Non-enzymatic H_2_S production uses cysteine as a specific substrate and requires coordinated catalysis by PLP and iron (Figure 1) [12]. Therefore, we decided to measure the bioavailability of molecules needed for non-enzymatic H_2_S synthesis in the placenta. Thus, the expression of several genes involved in the regulation of cellular iron homeostasis [30] was determined. However, gene expression of transferrin receptor-1 (TfR1), mitochondrial aconitase, and ferritin showed no differences between the three groups (Table S2).

**Figure 4:**
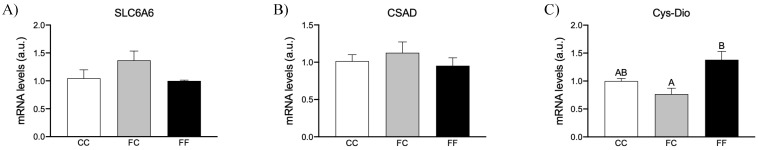
Maternal fructose intake affects placental cysteine bioavailability of their descendants. Placental levels of specific mRNA for (**A**) SLC6A6, (**B**) CSAD, and (**C**) Cys-Dio. Placental (mRNA) expression from control mothers (CC, empty bar), or pregnant rats from fructose-fed mothers subjected (FF, black bar) or not (FC, grey bar) to fructose intake throughout pregnancy. Data are expressed as means ± S.E., n = 5 (CC); 4 (FC); and 5 (FF) rats. Values not sharing a common letter are significantly different (*p* < 0.05). Relative target gene mRNA levels were measured by real-time PCR as explained in Materials and Methods, normalized to Rps29 levels and expressed in arbitrary units (a.u.). SLC6A6: solute carrier family 6 member 6; CSAD: cysteine sulfinic acid decarboxylase; Cys-Dio: cysteine dioxygenase.

Cys availability depends on its rate of synthesis through the transsulfuration process, and its catabolism by an oxidative pathway. As mentioned above, gene expression of enzymes involved in the transsulfuration pathway did not display differences between the experimental groups. Therefore, we focused on Cys catabolism. Cysteine is catabolized by cysteine dioxygenase (CysDio), which adds molecular oxygen to the sulfur atom of Cys, yielding cysteine sulfinate as a product. The obtained cysteine sulfinate can be decarboxylated to hypotaurine by cysteine sulfinate decarboxylase (CSAD), and then hypotaurine can be subsequently oxidized to taurine [31]. Thus, intracellular taurine levels are dependent on both the biosynthetic taurine enzymes CysDio and CSAD, and the taurine transporter encoded by the SLC6A6 gene. As shown in Figure 4, SLC6A6 (Figure 4A) and CSAD (Figure 4B) gene expression did not show any significant differences between the three groups of dams. However, placental CysDio gene expression, one of the most highly regulated metabolic enzymes known to respond to diet, was decreased in FC rats, becoming significant in comparison to FF mothers (Figure 4C, *p* = 0.012).

Therefore, the present findings suggest that placental non-enzymatic H_2_S production is augmented in pregnant rat descendants from fructose-fed mothers (FC) (Figure 3C), possibly due to a higher availability of Cys. In contrast, the decrease in non-enzymatic H_2_S synthesis found in these descendants when they consumed fructose (FF) could possibly be due to an increased catabolism of this sulfur amino acid.

### 3.3. Maternal Fructose Intake and Fructose Consumption Impact on Preeclampsia-Related Parameters

H_2_S plays a key role in vasodilation and angiogenesis during pregnancy. In fact, the involvement of H_2_S production in plasma levels of sFLT1, PGF, and other molecules related to preeclampsia has already been demonstrated. Nowadays, our diet has switched to a more Western-like diet, where fat and added sugars are predominant components. Preeclampsia is more prevalent in subjects with obesity or metabolic syndromes and in pregnant women who have a high intake of soft drinks [32]. In fact, we and others have previously found that maternal fructose intake affects placental metabolism, growth, and function [33,34]. Moreover, some authors have recently postulated that fructose might be a clue to the origin of preeclampsia [32]. This hypothesis proposed that fructose could contribute to the pathogenesis of preeclampsia by focusing on both the role of fructose production in the body (endogenous source) and the dietary intake of fructose (exogenous source) [32].

Given the effects reported regarding the role of H_2_S and fructose in preeclampsia, and since we have found that maternal fructose (FC) increases placental H_2_S synthesis in the offspring, whereas fructose intake (FF and fructose-fed dams) directly reduces H_2_S production, we can assume that fructose intake plays a key role in the development of preeclampsia.

Nakagawa et al. [32] related the possible involvement of fructose in preeclampsia with its metabolism and its consequent uric acid production. However, the gene expression of the three main enzymes of fructolysis: ketohexokinase, aldolase B, triokinase and FMN cyclase (TFKC) did not show any differences between the three groups (Table 1). Moreover, endogenous synthesis of fructose through the polyol pathway has also been related; however, aldose reductase (Table S2) and sorbitol dehydrogenase (Table 1) did not display significant differences between the three groups. Therefore, fructose metabolism did not seem to be affected.

It has been recently reported that fructose metabolism and hypoxia-inducible factor-1alpha (HIF1α) pathways are connected, affecting the development of various metabolic disorders. Indeed, many effects produced by HIF may be mediated by the stimulation of fructose production and metabolism [35,36]. Interestingly, since placental ischemia and hypoxia are typically found in preeclampsia, HIF1α has also been related to being involved in preeclampsia. Moreover, this hypoxia transcription factor does regulate fructose production and metabolism [32]. Thus, although gene expression of enzymes participating in the polyol pathway and fructose metabolism was not affected in the present study (Table 1), we studied mRNA gene expression of HIF1α and its target genes [36]. Neither the HIF1α target genes related to carbohydrate metabolism, such as glucose transporter 1; pyruvate dehydrogenase kinase 1; lactate dehydrogenase A; 6-Phosphofructo-2-Kinase/Fructose-2,6-Biphosphatase 3; and monocarboxylate transporter 1 (Table 1), nor other target genes, such as BCL2 interacting protein 3 and adenosine A2b receptor, showed significant differences between the three experimental groups. Curiously, the HIF1α target genes more related to preeclampsia, such as interleukin 1 β and vascular endothelial growth factor alpha, trended to be augmented, but not significantly, in both FC and FF dams compared to the control group (Table 1).

**Figure 5:**
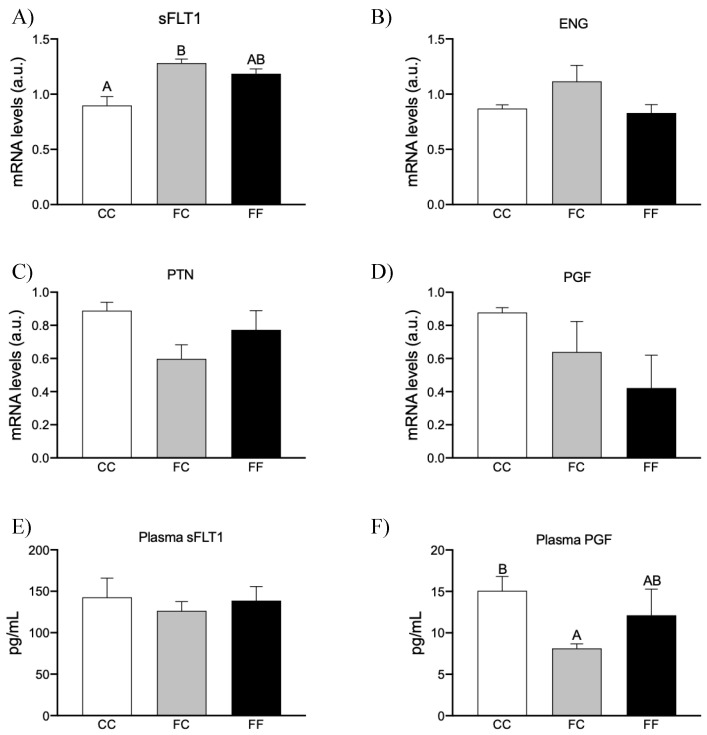
Maternal fructose intake influences placental molecules related to preeclampsia of their descendants. Placental levels of specific mRNA for (**A**) sFLT-1, (**B**) ENG, (**C**) PTN, and (**D**) PGF, and plasma levels of (**E**) sFLT-1 and (**F**) PGF. Placental (mRNA) expression and preeclampsia molecules levels from control mothers (CC, empty bar), or pregnant rats from fructose-fed mothers subjected (FF, black bar) or not (FC, grey bar) to fructose intake throughout pregnancy. Data are expressed as means ± S.E., n = 5 rats. Values not sharing a common letter are significantly different (*p* < 0.05). Relative target gene mRNA levels were measured by real-time PCR as explained in Materials and Methods, normalized to Rps29 levels and expressed in arbitrary units (a.u.). Data are expressed as means ± S.E., n = 5 (CC); 4 (FC); and 5 (FF) rats. Values not sharing a common letter are significantly different (*p* < 0.05). PTN: pleiotrophin; ENG: endoglin; PGF: placental growth factor; sFLT-1: soluble Fms-related receptor tyrosine kinase 1.

Taking these results into account, we determined if the production and release of preeclampsia-related proteins are affected by maternal fructose consumption and/or fructose intake. Further, since H_2_S has also been related to preeclampsia risk factors, we have also investigated the possible link between fructose consumption, H_2_S production, and preeclampsia-like conditions. Thus, as Figure 5 shows, whereas endoglin gene expression trended to be augmented in placenta of FC dams versus the other two groups, mRNA gene expression of sFLT1 (a well-known antiangiogenic factor) was augmented in both FC and FF mothers compared to CC rats, becoming significant in FC versus control dams (*p* = 0.032) (Figure 5A,B, respectively). These findings would indicate that maternal fructose intake promotes the production of these molecules with antiangiogenic properties in the placenta. According to these findings, placental gene expression of pleiotrophin (PTN) and PGF, two molecules with angiogenic properties [18,37], tended to be decreased by maternal fructose intake, although without reaching statistical significance (Figure 5C,D).

In addition, in contrast to placental sFLT1, whose gene expression was clearly augmented in FC and FF groups (Figure 5A), sFLT1 plasma levels remained unchanged in comparison to CC rats (Figure 5E). Interestingly, in accordance with placental PGF gene expression that tended to be decreased in both FC and FF dams (Figure 5D), plasma PGF was diminished, although it only became significant in pregnant rats from fructose-fed dams (FC) in comparison to CC rats (Figure 5F, *p* = 0.039). Curiously, Wang et al. (2013) [22] found that the release of PGF in placental explants is more sensitive to changes in H_2_S production than the release of sFLT1. Furthermore, expression of the sFLT1 gene has also been observed in tissues other than the placenta, such as in the lungs, kidneys, liver, and uterus [38], and this would explain the discrepancies observed between placental sFLT1 expression and its plasma levels. These findings would confirm that maternal fructose intake, subjected or not to a direct fructose consumption, induces the production and release of molecules involved in preeclampsia. However, these findings did not seem to be related to the data observed here in placental H_2_S synthesis (Figure 3) and they would be in contrast with those described by Wang et al. (2013) [22], since they found a link between the dysregulation of enzymatic placental H_2_S synthesis and the release of preeclampsia risk factors [22]. 

Indeed, these authors reported that the administration of a CSE inhibitor increased the release of plasma antiangiogenic factors (sFLT1 and endoglin), thereby increasing the risk of preeclampsia. Since the H_2_S gasotransmitter is not only produced in the placenta of pregnant rats and due to the fact that the liver is the main site of H_2_S production [39], we have also measured the hepatic production of H_2_S. Interestingly, hepatic H_2_S synthesis was more closely related to plasma levels of preeclamptic factors (Figure 5) because it was clearly decreased in FC and FF, although the differences did not become significant when compared to CC rats (21.26 ± 1.82, 15.78 ± 1.16, and 17.36 ± 1.16 nmol/g of tissue/min for CC, FC, and FF dams, respectively).

## 4. Discussion

It is striking that, in order to demonstrate the involvement of placental H_2_S in key processes of normal pregnancy [13,14] and in complications in gestation such as preeclampsia [15,22], most studies have only measured the expression of key enzymes in the transsulfuration pathway [16] or just determined the placental total production of H_2_S. Interestingly, we have demonstrated, for the first time and confirmed in two separate experimental models, that placental H_2_S is mainly non-enzymatically produced. This unexpected result would indicate that simply measuring placental CSE or CBS activities or even their protein levels to determine the involvement of placental H_2_S production in physiological or pathological pregnancies, such as preeclampsia [16,22,40], would not be sufficient. 

We have previously described that maternal fructose intake produces changes in female progeny that alter their own pregnancy, such as lipid accretion in the placenta [27]. However, in the present study we have found that maternal fructose consumption induces placental H_2_S synthesis in the offspring, when they are pregnant. Since H_2_S has shown a protective role against pathological pregnancies [41], it could be assumed that fructose consumption during gestation would be beneficial for the pregnancy of their descendants. Curiously, however, fructose intake directly leads to a diminution in placental H_2_S production, both if we compare pregnant rats from fructose-fed mothers receiving a fructose supplementation (FF) versus those that were not supplemented (FC), and fructose-fed versus glucose-fed dams. Therefore, this putative protective role of maternal fructose intake in H_2_S production could be beneficial in the pregnancy of the offspring, but not in their own gestation.

An angiogenic imbalance has been highlighted as the prime culprit in preeclampsia. Unexpectedly, maternal fructose intake was sufficient to significantly affect the synthesis and release of various factors involved in preeclampsia that would contribute to increasing the risk of developing this condition. Thus, in the present study, we have demonstrated that fructose induces the synthesis of antiangiogenic factors, sFlt1 and sEng, in the placenta, and reduces proangiogenic factors, as it decreases pleiotrophin production in the placenta and PGF synthesis and release into the plasma. Related to this, it is known that circulating levels of sFlt1, the endogenous inhibitor of VEGF and PGF, and sEng are elevated several weeks before the onset of the clinical manifestations of preeclampsia. Importantly, it has been demonstrated that VEGF plays a pivotal role in vasculogenesis and angiogenesis [38]. Furthermore, PGF is reduced in the first trimester of pregnant women who subsequently develop the syndrome [22].

Since pregnant rats descended from fructose-fed dams showed an increase in placental H_2_S production, it would indicate that H_2_S synthesis in the placenta is not always involved in preeclampsia. Curiously, the data found in the synthesis and release of preeclamptic factors would be more related to the results observed in the hepatic synthesis of H_2_S than those observed in the placenta. However, it should not be ruled out that placental H_2_S may be functioning as an antiangiogenic and/or vasoconstrictor factor, as it has been previously described for H_2_S in some situations. In fact, it has been suggested that the concentration/production rate of H_2_S, physiological/pathological conditions, and cell/tissue types used in the different studies could contribute to the divergent effects of H_2_S found on processes such as inflammatory responses and contraction versus relaxation of blood vessels [42].

Nevertheless, our study has some limitations that could be further addressed in future studies. Although several of the features that are characteristic of preeclampsia and proposed by Nakagawa et al. [32] were observed in both FC and FF dams, such as oxidative stress, insulin resistance, fatty liver, and increased placental uric acid [27], others such as reduced fetal weight or elevated liver enzymes were not found [27]. In addition, considering all the typical clinical manifestations of preeclampsia, such as hypertension, abnormal placental vascularization, proteinuria, endothelial dysfunction, and imbalance in angiogenic factors [22], only the latter parameter has been measured in the present study and has been shown to be affected in FC and FF dams. Therefore, to confirm that both fructose consumption and maternal fructose intake contribute to an increased risk of preeclampsia, more studies are needed. And, obviously, it is necessary to demonstrate that these findings that have been found here in experimental animals could be extrapolated to humans.

## 5. Conclusions

The present results would reinforce the importance of measuring placental non-enzymatic production of H_2_S (rather than total enzymatic production or only gene expression of transsulfuration enzymes) to elucidate the role of this gasotransmitter in pregnancy, under both physiological and pathological conditions. Moreover, regardless of the role of H_2_S in the risk of suffering preeclampsia, the negative influence of fructose consumption on the angiogenic imbalance and the consequent possible risk of developing preeclampsia is unquestionable. Therefore, a drastic reduction in the consumption of fructose-sweetened beverages, more importantly during pregnancy, should be urgently recommended.

## Figures and Tables

**Table 1:** Gene expression (mRNA) of enzymes related to fructose metabolism, polyol pathway, and HIF-1α target genes from placenta of control mothers (CC), or pregnant rats from fructose-fed mothers subjected (FF) or not (FC) to fructose intake throughout pregnancy.

	CC	FC	FF
Fructose Metabolism			
KHK	0.88 ± 0.10	1.76 ± 0.56	1.40 ± 0.207
Aldo B	1.86 ± 0.89	3.49 ± 2.35	0.88 ± 0.38
TKFC	1.00 ± 0.16	0.85 ± 0.19	0.78 ± 0.21
SDH	1.04 ± 0.17	1.10 ± 0.41	0.70 ± 0.04
HIF1α Target genes			
HIF1α	1.00 ± 0.05	1.42 ± 0.16	1.20 ± 0.12
GLUT1	1.16 ± 0.10	0.91 ± 0.02	0.92 ± 0.18
PDK1	0.92 ± 0.06	1.11 ± 0.07	1.20 ± 0.12
LDHA	0.99 ± 0.03	1.00 ± 0.10	0.87 ± 0.13
MCT1	1.02 ± 0.12	1.06 ± 0.19	0.81 ± 0.02
PFKB3	1.03 ± 0.15	1.04 ± 0.25	0.59 ± 0.07
BNIP3	0.99 ± 0.05	1.14 ± 0.11	1.07 ± 0.13
ADORA2B	1.02 ± 0.11	0.72 ± 0.11	0.91± 0.12
IL1β	0.82 ± 0.06	1.71 ± 0.44	1.61 ± 0.34
VEGF	0.90 ± 0.02	1.31 ± 0.02	1.25 ± 0.14

Data are expressed as means ± S.E., n = 5 (CC); 4 (FC); and 5 (FF) rats. Relative target gene mRNA levels were measured by real-time PCR as explained in Materials and Methods, normalized to Rps29 levels and expressed in arbitrary units (a.u.). KHK: ketohexokinase; Aldo B: aldolase B; TKFC: triokinase and FMN cyclase; SDH: sorbitol dehydrogenase; HIF-1α: hypoxia-inducible factor 1 subunit alpha; GLUT1: glucose transporter; PDK1: pyruvate dehydrogenase kinase 1; LDHA: lactate dehydrogenase A; MCT1: monocarboxylate transporter 1; BNIP3: BCL2 interacting protein 3; ADORA2B: adenosine A2b receptor; PFKB3: 6-Phosphofructo-2-Kinase/Fructose-2,6-Biphosphatase 3; IL1β: interleukin 1 β; VEGF: vascular endothelial growth factor.

## Data Availability

All data generated or analyzed during this study are available from the corresponding author on reasonable request. The data are not publicly available due to principle of confidentiality.

## Supplementary Materials

The following supporting information can be downloaded at: https://www.mdpi.com/article/10.3390/nu16020309/s1, Table S1: Placental gene expression in pregnant rats subjected to fructose or glucose intake throughout pregnancy and control mothers; and Table S2: Placental gene expression in pregnant rats from fructose-fed mothers subjected (FF) or not (FC) to fructose intake throughout pregnancy and control mothers.

## References

[1] Carvallo P., Carvallo E., Barbosa-da-Silva S., Mandarim-de-Lacerda C.A., Hernández A., del-Sol M. (2019). Efectos metabólicos del con-sumo excesivo de fructosa añadida. Int. J. Morphol..

[2] Khitan Z., Kim D.H. (2013). Fructose: A Key Factor in the Development of Metabolic Syndrome and Hypertension. J. Nutr. Metab..

[3] Dupas J., Feray A., Goanvec C., Guernec A., Samson N., Bougaran P., Guerrero F., Mansourati J. (2017). Metabolic Syndrome and Hypertension Resulting from Fructose Enriched Diet in Wistar Rats. BioMed Res. Int..

[4] Delbosc S., Paizanis E., Magous R., Araiz C., Dimo T., Cristol J.P., Cros G., Azay J. (2005). Involvement of oxidative stress and NADPH oxidase ac-tivation in the development of cardiovascular complications in a model of insulin resistance, the fructose-fed rat. Atherosclerosis.

[5] Johnson R.J., Segal M.S., Sautin Y., Nakagawa T., Feig D.I., Kang D.-H., Gersch M.S., Benner S., Sánchez-Lozada L.G. (2007). Potential role of sugar (fructose) in the epidemic of hypertension, obesity and the metabolic syndrome, diabetes, kidney disease, and cardiovascular disease. Am. J. Clin. Nutr..

[6] Hannou S.A., Haslam D.E., McKeown N.M., Herman M.A. (2018). Fructose metabolism and metabolic disease. J. Clin. Investig..

[7] Hocher B. (2007). Fetal programming of cardiovascular diseases in later life–mechanisms beyond maternal undernutrition. J. Physiol..

[8] Marciniak A., Patro-Małysza J., Kimber-Trojnar Ż., Marciniak B., Oleszczuk J., Leszczyńska-Gorzelak B. (2017). Fetal programming of the metabolic syndrome. Taiwan. J. Obstet. Gynecol..

[9] Perrone S., Santacroce A., Picardi A., Buonocore G. (2016). Fetal programming and early identification of newborns at high risk of free radical-mediated diseases. World J. Clin. Pediatr..

[10] Wallace J.L., Blackler R.W., Chan M.V., Da Silva G.J., Elsheikh W., Flannigan K.L., Gamaniek I., Manko A., Wang L., Motta J.-P. (2015). Anti-inflammatory and cytoprotective actions of hydrogen sulfide: Translation to therapeutics. Antioxid. Redox Signal..

[11] Finkelstein J.D., Martin J.J. (2000). Homocysteine. Int. J. Biochem. Cell Biol..

[12] Yang J., Minkler P., Grove D., Wang R., Willard B., Dweik R., Hine C. (2019). Non-enzymatic hydrogen sulfide production from cysteine in blood is catalyzed by iron and vitamin B6. Commun. Biol..

[13] Liu W., Xu C., You X., Olson D.M., Chemtob S., Gao L., Ni X. (2016). Hydrogen Sulfide Delays LPS-Induced Preterm Birth in Mice via Anti-Inflammatory Pathways. PLoS ONE.

[14] You X.-J., Xu C., Lu J.-Q., Zhu X.-Y., Gao L., Cui X.-R., Li Y., Gu H., Ni X. (2011). Expression of cystathionine β-synthase and cystathionine γ-lyase in human pregnant myometrium and their roles in the control of uterine contractility. PLoS ONE.

[15] Holwerda K.M., Faas M.M., van Goor H., Lely A.T. (2013). Gasotransmitters: A solution for the therapeutic dilemma in preeclampsia?. Hypertension.

[16] Holwerda K.M., Bos E.M., Rajakumar A., Ris-Stalpers C., van Pampus M.G., Timmer A., Erwich J.J.H.M., Faas M.M., van Goor H., Lely A.T. (2012). Hydrogen sulfide producing enzymes in pregnancy and preeclampsia. Placenta.

[17] Ghulmiyyah L., Sibai B. (2012). Maternal Mortality From Preeclampsia/Eclampsia. Semin. Perinatol..

[18] Phipps E.A., Thadhani R., Benzing T., Karumanchi S.A. (2019). Pre-eclampsia: Pathogenesis, novel diagnostics and therapies. Nat. Rev. Nephrol..

[19] Levine R.J., Maynard S.E., Qian C., Lim K.-H., England L.J., Yu K.F., Schisterman E.F., Thadhani R., Sachs B.P., Epstein F.H. (2004). Circulating angiogenic factors and the risk of preeclampsia. N. Engl. J. Med..

[20] Park J.E., Chen H.H., Winer J., Houck K.A., Ferrara N. (1994). Placenta growth factor. Potentiation of vascular endothelial growth factor bioactivity, in vitro and in vivo, and high affinity binding to Flt-1 but not to Flk-1/KDR. J. Biol. Chem..

[21] Rengarajan A., Mauro A.K., Boeldt D.S. (2020). Maternal disease and gasotransmitters. Nitric Oxide.

[22] Wang K., Ahmad S., Cai M., Rennie J., Fujisawa T., Crispi F., Ahmed A. (2013). Dysregulation of hydrogen sulfide producing enzyme cysta-thionine γ-lyase contributes to maternal hypertension and placental abnormalities in preeclampsia. Circulation..

[23] Wang R. (2023). Roles of Hydrogen Sulfide in Hypertension Development and Its Complications: What, So What, Now What. Hypertension.

[24] Fauste E., Rodrigo S., Aguirre R., Donis C., Rodríguez L., Álvarez-Millán J.J., Panadero M.I., Otero P., Bocos C. (2020). Maternal Fructose Intake Increases Liver H_2_S Synthesis but Exarcebates its Fructose-Induced Decrease in Female Progeny. Mol. Nutr. Food Res..

[25] Roglans N., Fauste E., Bentanachs R., Velázquez A.M., Pérez-Armas M., Donis C., Laguna J.C. (2022). Bempedoic Acid Restores Liver H2S Pro-duction in a Female Sprague-Dawley Rat Dietary Model of Non-Alcoholic Fatty Liver. Int. J. Mol. Sci..

[26] Rodríguez L., Panadero M.I., Roglans N., Otero P., Alvarez-Millán J.J., Laguna J.C., Bocos C. (2013). Fructose during preg-nancy affects maternal and fetal leptin signaling. J. Nutr. Biochem..

[27] Fauste E., Panadero M.I., Donis C., Otero P., Bocos C. (2021). Pregnancy Is Enough to Provoke Deleterious Effects in Descendants of Fructose-Fed Mothers and Their Fetuses. Nutrients.

[28] Hine C., Mitchell J.R. (2017). Endpoint or Kinetic Measurement of Hydrogen Sulfide Production Capacity in Tissue Extracts. Bio-protocol.

[29] Pfaffl M.W. (2001). A new mathematical model for relative quantification in real-time RT-PCR. Nucleic Acids Res..

[30] Hentze M.W., Muckenthaler M.U., Andrews N.C. (2004). Balancing Acts: Molecular Control of Mammalian Iron Metabolism. Cell.

[31] Stipanuk M.H., Ueki I. (2011). Dealing with methionine/homocysteine sulfur: Cysteine metabolism to taurine and inorganic sulfur. J. Inherit. Metab. Dis..

[32] Nakagawa T., Andres-Hernando A., Kosugi T., Sanchez-Lozada L.G., Stenvinkel P., Kublickiene K., Karumanchi S.A., Kang D.-H., Kojima H., Rodriguez-Iturbe B. (2023). Fructose might be a clue to the origin of preeclampsia insights from nature and evolution. Hypertens. Res..

[33] Rodrigo S., Rodríguez L., Otero P., Panadero M.I., García A., Barbas C., Roglans N., Ramos S., Goya L., Laguna J.C. (2016). Fructose during pregnancy provokes fetal oxidative stress: The key role of the placental heme oxygenase-1. Mol. Nutr. Food Res..

[34] Vickers M.H., Clayton Z.E., Yap C., Sloboda D.M. (2011). Maternal fructose intake during pregnancy and lactation alters placental growth and leads to sex-specific changes in fetal and neonatal endocrine function. Endocrinology..

[35] Kanbay M., Altıntas A., Yavuz F., Copur S., Sanchez-Lozada L.G., Lanaspa M.A., Johnson R.J. (2023). Responses to Hypoxia: How Fructose Me-tabolism and Hypoxia-Inducible Factor-1a Pathways Converge in Health and Disease. Curr. Nutr. Rep..

[36] Eberhart T., Schönenberger M.J., Walter K.M., Charles K.N., Faust P.L., Kovacs W.J. (2020). Peroxisome-Deficiency and HIF-2α Signaling Are Negative Regulators of Ketohexokinase Expression. Front. Cell Dev. Biol..

[37] Perez-Pinera P., Berenson J.R., Deuel T.F. (2008). Pleiotrophin, a multifunctional angiogenic factor: Mechanisms and pathways in normal and pathological angiogenesis. Curr. Opin. Hematol..

[38] He Y., Smith S.K., Day K.A., Clark D.E., Licence D.R., Charnock-Jones D.S. (1999). Alternative splicing of vascular endothelial growth factor (VEGF)-R1 (FLT-1) pre-mRNA is important for the regulation of VEGF activity. Mol. Endocrinol..

[39] Patel P., Vatish M., Heptinstall J., Wang R., Carson R.J. (2009). The endogenous production of hydrogen sulphide in intrauterine tissues. Reprod. Biol. Endocrinol..

[40] Cindrova-Davies T., Herrera E.A., Niu Y., Kingdom J., Giussani D.A., Burton G.J. (2013). Reduced cystathionine γ-lyase and increased miR-21 expression are associated with increased vascular resistance in growth-restricted pregnancies: Hydrogen sulfide as a placental vasodilator. Am. J. Pathol..

[41] Zhang Y.-X., Jing M.-R., Cai C.-B., Zhu S.-G., Zhang C.-J., Wang Q.-M., Zhai Y.-K., Ji X.-Y., Wu D.-D. (2023). Role of hydrogen sulphide in physiological and pathological angiogenesis. Cell Prolif..

[42] Li L., Moore P.K. (2008). Putative biological roles of hydrogen sulfide in health and disease: A breath of not so fresh air?. Trends Pharmacol. Sci..

