# Supplementary material for: Fructose Consumption Affects Placental Production of H2S: Impact on Preeclampsia-Related Parameters
Source: Nutrients. 2024 Jan 20;16(2):309. doi: 10.3390/nu16020309 (PMC10820116; doi:10.3390/nu16020309)
Supplement: Supplementary file 1 [file nutrients-16-00309-s001.zip › nutrients-2759263-supplementary.pdf]

Table S1. Placental gene expression in pregnant rats subjected to fructose or glucose intake throughout pregnancy and control mothers.

	CONTROL	FRUCTOSE	GLUCOSE
**CSE (a.u.)**	0.069 ± 0.011	0.097 ± 0.013	0.099 ± 0.023
**CBS (a.u.)**	0.008 ± 0.001	0.023 ± 0.008	0.005 ± 0.000
**3MST (a.u.)**	0.715 ± 0.033	0.793 ± 0.092	0.672 ± 0.046

Data are expressed as means ± S.E., n= 7 (control); 7 (fructose); and 6 (glucose) rats. Relative target gene mRNA levels were measured by real-time PCR as explained in Materials and Methods, normalized to Rps29 levels and expressed in arbitrary units (a.u.). CSE: Cystathionine-γ-lyase; CBS: Cystathionine-β-synthase; 3MST: 3-Mercaptopyruvate Sulfotransferase.

Table S2. Placental gene expression in pregnant rats from fructose-fed mothers subjected (FF) or not (FC) to fructose intake throughout pregnancy and control mothers.

	CC	FC	FF
**CSE (a.u.)**	0.068 ± 0.004	0.077 ± 0.011	0.062 ± 0.004
**CBS (a.u.)**	0.005 ± 0.001	0.004 ± 0.0004	0.004 ± 0.0007
**3MST (a.u.)**	0.96 ± 0.026	1.02 ± 0.06	1.11 ± 0.07
**ACO1 (a.u.)**	0.98 ± 0.013	1.20 ± 0.24	1.04 ± 0.09
**ACO2 (a.u.)**	0.94 ± 0.022	1.06 ± 0.15	1.036 ± 0.092
**Ferritin (a.u.)**	1.019 ± 0.10	1.15 ± 0.204	1.024 ± 0.08
**TFR1 (a.u.)**	1.027 ± 0.11	1.14 ± 0.33	0.83 ± 0.14
**AR (a.u.)**	36.16 ± 2.39	29.85 ± 2.61	29.52 ± 1.54

Placental (mRNA) expression from control mothers (CC), or pregnant rats from fructose-fed mothers subjected (FF) or not (FC) to fructose intake throughout pregnancy. Data are expressed as means ± S.E., n = 5 (CC); 4 (FC); and 5 (FF) rats. Relative target gene mRNA levels were measured by real-time PCR as explained in Materials and Methods, normalized to Rps29 levels and expressed in arbitrary units (a.u.). CSE: Cystathionine-γ-lyase; CBS: Cystathionine-β-synthase; 3MST: 3-Mercaptopyruvate Sulfotransferase, ACO1: Aconitase 1; ACO2: Aconitase 2; TFR1: Transferrin Receptor 1; AR: Aldose reductase.
